# Helix encoder: a compound-protein interaction prediction model specifically designed for class A GPCRs

**DOI:** 10.3389/fbinf.2023.1193025

**Published:** 2023-05-26

**Authors:** Haruki Yamane, Takashi Ishida

**Affiliations:** Department of Computer Science, School of Computing, Tokyo Institute of Technology, Tokyo, Japan

**Keywords:** compound-protein interaction, class A GPCR, deep learning, ligand binding site, transmembrane region, extracellular loop

## Abstract

Class A G protein-coupled receptors (GPCRs) represent the largest class of GPCRs. They are essential targets of drug discovery and thus various computational approaches have been applied to predict their ligands. However, there are a large number of orphan receptors in class A GPCRs and it is difficult to use a general protein-specific supervised prediction scheme. Therefore, the compound-protein interaction (CPI) prediction approach has been considered one of the most suitable for class A GPCRs. However, the accuracy of CPI prediction is still insufficient. The current CPI prediction model generally employs the whole protein sequence as the input because it is difficult to identify the important regions in general proteins. In contrast, it is well-known that only a few transmembrane helices of class A GPCRs play a critical role in ligand binding. Therefore, using such domain knowledge, the CPI prediction performance could be improved by developing an encoding method that is specifically designed for this family. In this study, we developed a protein sequence encoder called the Helix encoder, which takes only a protein sequence of transmembrane regions of class A GPCRs as input. The performance evaluation showed that the proposed model achieved a higher prediction accuracy compared to a prediction model using the entire protein sequence. Additionally, our analysis indicated that several extracellular loops are also important for the prediction as mentioned in several biological researches.

## 1 Introduction

G protein-coupled receptors (GPCRs) are seven-transmembrane proteins that are activated by various ligands, such as hormones, neurotransmitters, and sensory stimuli (Di Pizio et al., 2019). Due to their characteristics, GPCRs are involved in many diseases, and approximately 35% of approved drugs target GPCRs (Sriram and Insel, 2018). This is because GPCRs are expressed on the cell membrane, which facilitates molecular interactions in the extracellular environment, and because their binding sites for compounds are well-defined (Di Pizio et al., 2019). GPCRs preserve a signal transduction mechanism that involves a large conformational change to fit with G proteins. This mechanism is encoded by conserved motifs found throughout all seven transmembrane domains (TMs) and forms a TM-interacting network that converges on the cytoplasmic side (Cong et al., 2017). Specifically, when the 7 TMs are numbered as TM1 to TM7 starting from the N-terminus, motifs such as “D(E)RY” in TM3, “CWLP” in TM6, and “NPxxY“ in TM7 are present (Venkatakrishnan et al., 2013). GPCR is classified into six classes based on sequence similarity: class A, rhodopsin-like; class B, secretin-like; class C, metabotropic glutamate receptor-like; class D, fungal pheromone receptor-like; class E, cAMP receptor-like; and class F, frizzled/smoothened-like (Davies et al., 2008; Harding et al., 2017). While having 7 TMs is a common feature among all classes of GPCRs, each class has specific structural features, such as the highly complex and elongated N-terminus in class B and C GPCRs (compared to class A GPCRs), including a Venus flytrap domain in class C GPCRs. This results in differences in the binding regions of compounds among GPCR classes. In class A GPCRs, the binding region of compounds is only the seven-transmembrane domains. However, in class B GPCRs and class C GPCRs, the very long N-terminal domains also become binding regions for compounds (Di Pizio et al., 2019). Class A GPCRs are the largest subfamily of human GPCRs, including rhodopsin, adrenergic receptors, and olfactory receptors. These proteins are known to have a ligand-binding site in the alpha helix region of the transmembrane domain (Venkatakrishnan et al., 2013). Many orphan receptors are present in class A GPCRs. Therefore, deorphanization of class A GPCRs is considered to be very important for drug discovery. Studies on predicting the binding of compounds to class A GPCRs have long been conducted using structure-based and ligand-based virtual screening techniques. Target proteins include adenosine receptors (Carlsson et al., 2010; Wei et al., 2020; Jacobson et al., 2022), adrenaline receptors (Sabio et al., 2008; Kolb et al., 2009; Chevillard et al., 2019), chemokine receptors (Mysinger et al., 2012; Mishra et al., 2016; Adlere et al., 2019), and olfactory receptors (Ahmed et al., 2018; Yuan et al., 2019), and these research have contributed to the discovery of novel ligands. However, structure-based virtual screening requires highly accurate 3D models of proteins, which is difficult to be applied to proteins with unknown structures such as olfactory receptors. Ligand-based virtual screening, on the other hand, is effective for proteins with sufficient ligand information and is not suitable for proteins with limited ligand information. Furthermore, as mentioned earlier, class A GPCRs have a high prevalence of orphan receptors, making ligand-based methods unsuitable.

As a solution to this problem, there is a method of predicting CPI (compound-protein interaction) using machine learning from the protein sequence information and compound structure information (Bleakley and Yamanishi, 2009; van Laarhoven et al., 2011; Cheng et al., 2012; Wang and Zeng, 2013). This approach has been applied to various protein families, and deep learning-based predictions have been shown to be effective. Several deep learning models, such as DeepDTA (Öztürk et al., 2018), which encodes protein sequences and compounds and extracts features using convolutional neural networks, CPI-GNN (Tsubaki et al., 2018) and GraphDTA (Nguyen et al., 2019), which use graph neural networks instead of convolutional neural networks, have been proposed. In recent years, a CPI prediction model called TransformerCPI (Chen et al., 2020), which utilizes Transformer (Vaswani et al., 2017) and was specifically designed for the CPI prediction task, was proposed.

When applying existing CPI prediction models to class A GPCRs, the prediction accuracy is insufficient and there is room for improvement. Recent protein sequence-based CPI prediction models use the entire protein sequence as input information for the protein side, in order to make them applicable to various protein families. However, protein residues in the sequence include not only those directly related to binding but also those that are not involved in binding, such as the intrinsically disordered regions at the N- and C-termini, etc. If the entire protein sequence is encoded as input, regions that are not directly related to binding are also encoded. Non-binding regions can become noise and potentially degrade the model’s predictive performance.

According to previous studies, it has been reported that performance can be improved by targeting a specific protein family and limiting the protein sequence used in the prediction model to only the important parts for ligand binding, rather than the entire sequence (Chepurwar et al., 2019; Cong et al., 2022; Lee and Nam, 2022). Cong et al. identified important protein residues involved in ligand binding based on docking simulations of a limited number of odorant receptor-compound pairs, targeting the olfactory receptor which is a type of class A GPCR(Cong et al., 2022). However, it cannot be concluded that these residues are important for all proteins in the dataset. In addition, Ingoo et al. obtained ligand binding regions from 3D complexes (Lee and Nam, 2022). This method is only applicable to proteins with known 3D structures and is not suitable for families that include proteins with unknown 3D structures, such as class A GPCRs. Therefore, it is difficult to identify the optimal protein residues involved in ligand binding that are common to all class A GPCRs. However, as previously mentioned, it is known that the ligand binding region of class A GPCRs is located in the transmembrane helices. Thus, it is possible to select important input protein residues for ligand binding based on this domain knowledge.

To address the problem, we proposed a class A GPCR-specific encoding model called a Helix encoder. It focuses amino acid sequence of seven transmembrane helix regions of class A GPCRs and uses them as the input. By replacing the encoder part of TransformerCPI with the Helix encoder, we developed a class A GPCR-specific CPI prediction model. We also constructed a dataset of compound-protein interaction information of class A GPCRs. We evaluated the performance of our proposed class A GPCR-specific CPI prediction model using the dataset and compared the performance with a CPI prediction model which uses a whole protein sequence as the input.

## 2 Materials and methods

### 2.1 Dataset construction

We constructed a dataset only including compound-protein interaction information of class A GPCRs. GLASS DB (Chan et al., 2015) was used to construct the class A GPCR dataset. This database contains experimentally validated information on the interaction between GPCRs and ligands, with 743,031 interaction information for 707 proteins and 316,814 compounds. To extract the information of class A GPCRs from the database, GPCRdb (Pándy-Szekeres et al., 2022) was referred. We used UniProt (Bateman et al., 2021) IDs to determine whether the protein was a class A GPCR. In this study, we deal with the activity prediction problem as a binary classification of whether a target protein is activated by the ligand or not. We only used affinity data evaluated by IC50 or EC50. If the negative logarithms of the affinity value were 6 or more, we labeled it as positive. Additionally, as pointed out in the TransformerCPI study on ligand bias, compounds with only one interaction were removed, as were compounds that existed only in one class. The final class A GPCR dataset is described in the Table1.

**TABLE 1:** Details of the class A GPCR dataset.

Proteins	Compounds	Interactions	Positive	Negative
382	11,246	31,888	15,801	16,708

### 2.2 Input features

As we mentioned, basically only the transmembrane helix sequences involved in binding with compounds in class A GPCR. Thus, we only used the transmembrane regions as the input of the proposed model. Protein sequence information was obtained from UniProt. At that time, the start and end positions of each helix were obtained from the secondary structure information registered in UniProt, and the protein sequence was divided into seven helix sequences. The word2vec (Mikolov et al., 2013) algorithm used in TransformerCPI was used to encode each helix sequence. Finally, the protein sequence input becomes a *p* × 100 dimensional feature vector, where *p* is the length of each helix sequence.

We used the same embedding method for compounds used in TransformerCPI. The compounds in the class A GPCR dataset were represented by canonical SMILES, and each atom was converted to a 34-dimensional feature vector using RDKit. Furthermore, the representation of each atom, which integrates the features of neighboring atoms using graph neural networks, was learned, and the input format was a*a* × 64 dimensional feature vector, where *a* is the number of atoms.

### 2.3 Helix encoder

**FIGURE 1:**
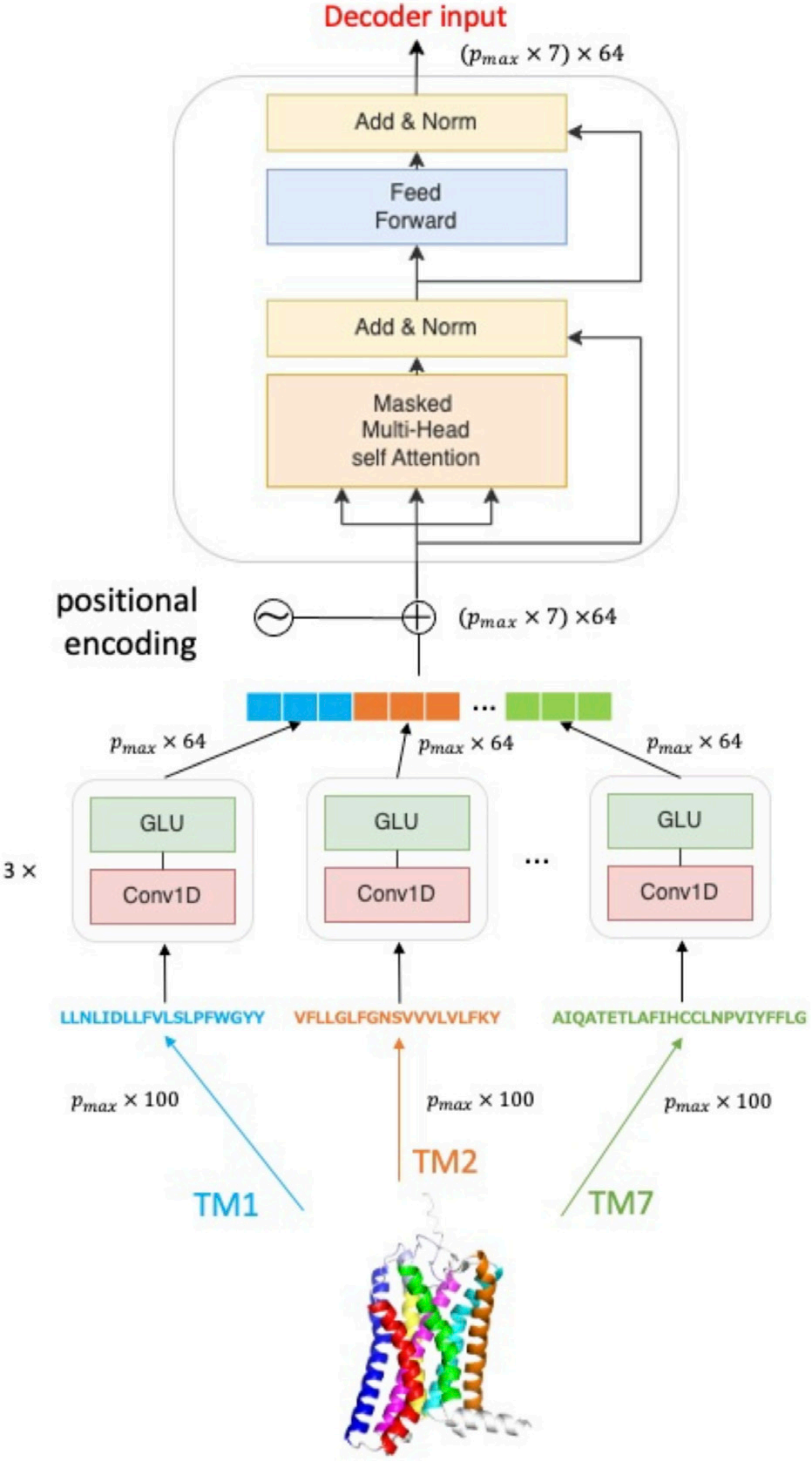
Overview of Helix encoder. The amino acid sequence of a class A GPCR protein is divided into seven subsequences (TM1, TM2, …TM7) according to transmembrane region information and then they are independently processed in the encoding unit.

An overview of the Helix encoder is given in Figure 1. The architecture of the Helix encoder consists of two blocks: one block composed of a 1D convolution layer and a gated linear unit (GLU) layer (Dauphin et al., 2017), and another block composed of a multi-headed attention layer and a feedforward layer that forms a self-attention block. The helix feature vectors, which are embedded into *p* × 100 dimensions by word2vec, are first inputted into the block consisting of the 1D convolution layer and GLU. At this point, the maximum length *p* *_max_* among the length *p*_*i*_ (*i* = 1, 2, …, 7) of each helix is taken, and zero-padding is performed for helices shorter than *p* *_max_*. This results in all helix feature vectors becoming *p* *_max_* × 100 dimensions, which then serve as input. The block consisting of the 1D convolution layer and GLU layer adopts the same architecture as the protein sequence encoder of TransformerCPI. The helix feature vectors encoded by each 1D convolution layer and GLU layer are concatenated and subjected to positional encoding. The protein sequence vector, which is positional encoded, becomes (*p* *_max_* × 7) × 64 dimensions, and serves as input to the self-attention block. The output of the multi-headed attention layer is added to the input value and then normalized by layer normalization, which serves as input to the feedforward layer. The final output is a (*p* *_max_* × 7) × 64 dimensional feature vector, which serves as input to the decoder of TransformerCPI. Details of the hyperparameters are listed in Supplementary Table S1.

### 2.4 Evaluation

This study treats class A GPCR activity prediction as a binary classification problem. The receiver operating characteristic (ROC) curves were used for analyzing the performance and the area under the ROC curve (AUC) was used as the performance evaluation metric. Two models were constructed based on a TransformerCPI model in this experiment: one replaced the sequence encoder with a Helix encoder that limited input sequences to transmembrane helices, while the other used the sequence encoder using whole sequences as the input (same as the original TransformerCPI’s encoder). The class A GPCR dataset was randomly split into training and test data in an 8:2 ratio for training and evaluation, respectively. The data was split randomly, so multiple split patterns could be created by changing the seed value. Therefore, in this study, five patterns of training and test data were prepared, i.e., (train_0_, test_0_), …, (train_4_, test_4_). The statistical significance of the improvement was confirmed based on them. The training data were randomly split into training and validation data in an 8:2 ratio, and the final model was selected based on the validation data. All models were evaluated and compared based on the AUC of the test data for the final model with the highest AUC based on the validation data during 100 epochs.

## 3 Results

### 3.1 Training detail

The learning curves of the TransformerCPI encoder model and Helix encoder model are shown in Supplementary Figure S1, S2. Both models show a decrease in the training loss over 100 epochs, but the validation AUC has started to converge within the first 100 epochs. Across all validation cases, the maximum AUC based on the validation data occurs between 80 and 100 epochs, and the model at that point is selected as the final model. The average AUC based on the five validation datasets is 0.918 for the Helix encoder model and 0.911 for the TransformerCPI encoder model.

### 3.2 Model performance

The model using the Helix encoder achieved a higher AUC than the TransformerCPI encoder model for both validation and test data. Table 2 shows the performance of both models on the five test cases. A one-sided paired *t*-test with a significance level of 5% was performed on the AUCs for the five test cases, showing that the performance improvement of the Helix encoder is significant (*p* = 0.0015 < 0.05) and confirming that the Helix encoder is effective in predicting the activity of class A GPCRs.

**TABLE 2:** Prediction accuracy in test cases (AUC).

Model	test_0_	test_1_	test_2_	test_3_	test_4_	Average
TransformerCPI encoder	0.912	0.912	0.916	0.910	0.916	0.913
Helix encoder	0.922	0.920	0.919	0.920	0.922	0.920

**FIGURE 2:**
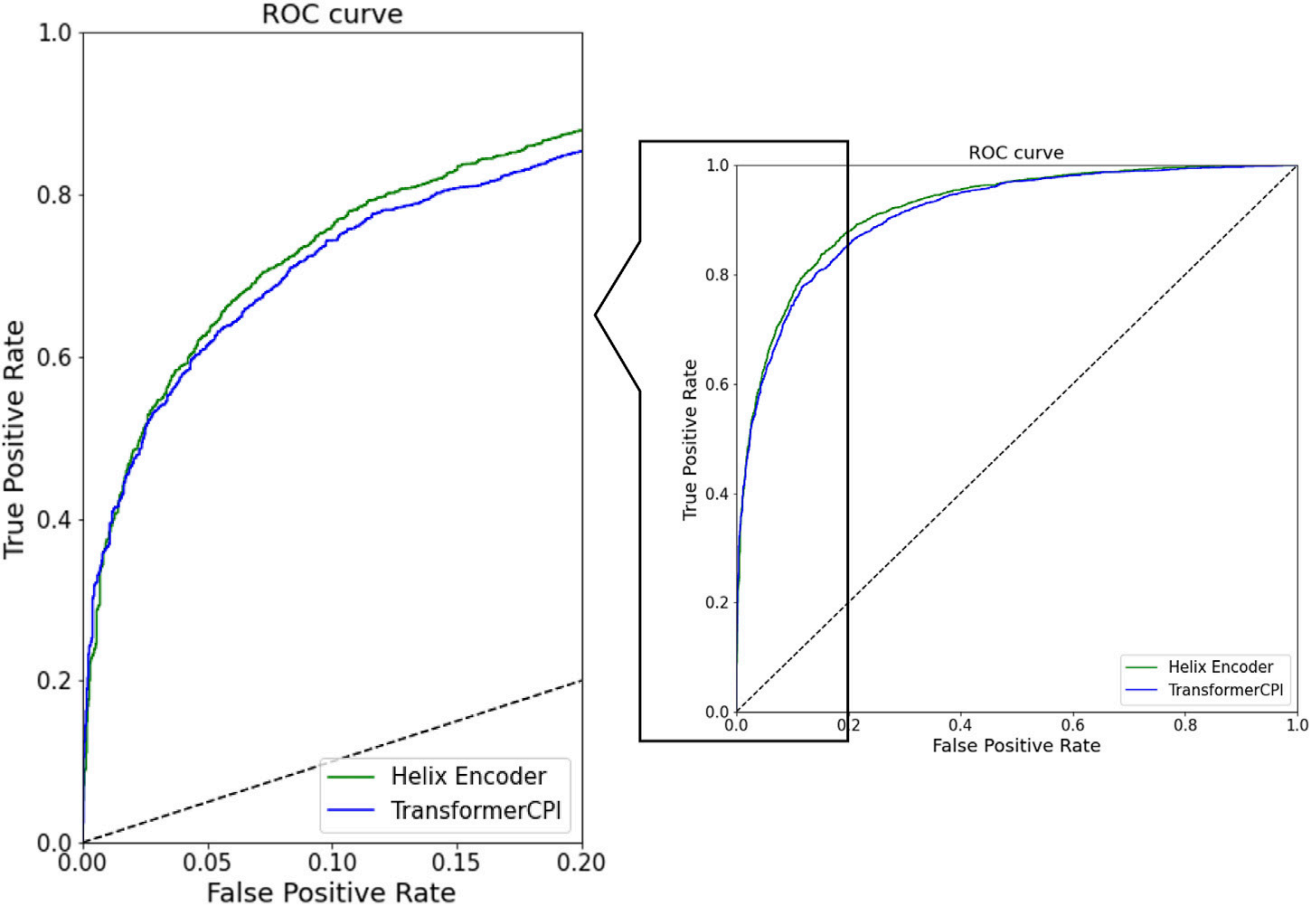
ROC curve of prediction results of Helix encoder and TransformerCPI encoder. The green curve is the Helix encoder model and the blue curve is the TransformerCPI encoder model.

The ROC curves for both models are shown in Figure 2. The ROC curve of the Helix encoder exhibits a larger curve than that of the TransformerCPI encoder model. Specifically, at the low false positive rate stage (false positive rate = 0.2), the Helix encoder achieves a higher true positive rate than the TransformerCPI encoder. In contrast, the Helix encoder shows comparable or worse accuracy with a false positive rate of less than 0.05. It is probably because prediction can be made based only on the ligand information for several cases, and the improvement of protein-sequence encoding may not have a significant impact on such cases.

### 3.3 Performance for different receptor subfamilies

Class A GPCRs consist of several subfamilies, such as aminergic receptors, peptide receptors, etc. We calculated the prediction accuracy (AUC) for each subfamily using the subfamily definition of the GPCRdb database (Pándy-Szekeres et al., 2022). The summaries of the results are shown in the Table 3. Several subfamilies, such as melatonin receptors, have a limited number of data and the prediction accuracy was much lower than a subfamily with sufficient data. We hoped that the CPI predictions would maintain accuracy even for smaller subfamilies, but it turns out that sufficient information about closely related proteins is still important.

**TABLE 3:** Prediction accuracy for different subfamilies.

Subfamily	#Interactions	AUC
Aminergic receptors	8294	0.846
Peptide receptors	10,719	0.925
Protein receptors	470	0.930
Lipid receptors	8494	0.971
Melatonin receptors	29	0.738
Nucleotide receptors	1237	0.874
Steroid receptors	68	0.805
Alicarboxylic acid receptors	81	0.847
Orphan receptors	144	0.704

### 3.4 Transferability of a prediction model and the performance for novel ligands

This study uses random splitting to divide the test and training data. Therefore, many proteins included in the test data are also included in the training set, making it inappropriate for estimating prediction accuracy for novel proteins. Therefore, proteins in the test set that are not included in the training set were extracted from the cross-validation results. As a result, 37 proteins were extracted. The prediction accuracy (AUC) for the subset was 0.786 and it is much worse than that for the remaining cases (the target protein of the test set is included in the training data set). Unfortunately, this indicates that the transferability of the proposed method is still insufficient.

We also checked the prediction performance of the proposed method for novel ligands. We checked the oldest publication year of each ligand using the ChEMBL literature record. As a result, 872 out of 11,246 ligands were published after 2015. For the novel ligands, we calculated the prediction accuracy and the AUC was 0.825. We considered that the lower accuracy was due to the low similarity of such novel ligands to the old ones. Therefore, we calculated the average Tanimoto similarity of the ECFP4 fingerprint between the novel and old ones and within the novel ones, but we could not find clear differences (0.334 and 0.353, respectively). The reason for the poor performance against novel ligands may be due to more complex compound structures.

### 3.5 Comparison with docking simulation

Docking simulation is one of the main methods in structure-based virtual screening. However, a direct performance comparison between the proposed method and docking simulation is difficult because many proteins do not have an experimentally determined 3D structure. Therefore, we selected a protein (UniProt ID: P42866) with the most interaction data among the proteins with known 3D structures in our dataset and compared the performance of the proposed method and docking simulation for the data. We performed the docking simulation using Autodock Vina ver 1.2 (Trott and Olson, 2010). The center coordinates of the search box were manually set using ChimeraX, and $boxsize=20\overset{{}^{\circ}}{A}\times 20\overset{{}^{\circ}}{A}\times 20\overset{{}^{\circ}}{A}$ was used. As a result, the prediction accuracy (AUC) of the docking simulation was 0.766. In contrast, the proposed method showed AUC = 0.882 and was much better. However, this target protein has enough interactions (925 interactions), so the setting is more favorable for CPI prediction. As mentioned above, the prediction accuracy of the proposed method for the novel target protein was less than 0.8. Therefore, the prediction accuracies of the two methods would be almost comparable for such a situation.

## 4 Discussion

### 4.1 Influence of extracellular loop

#### 4.1.1 Extracellular loop 2

As mentioned earlier, class A GPCRs form a ligand-binding pocket within their seven transmembrane domains for interaction with compounds. However, it has been reported that there are proteins that have residues that directly interact with certain compounds when binding to the extracellular loop 2 (ECL2) (Wheatley et al., 2012). Adding ECL2 as an input feature may therefore improve the accuracy of activity prediction. ECL2 is an extracellular loop located between transmembrane domains 4 and 5 (TM4 to TM5) of GPCRs, counted from the N-terminal region of the transmembrane domain. ECL2 has a very long and highly diversified sequence compared to other extracellular loops (Wheatley et al., 2012; Wolf and newald, 2015). On the other hand, the disulfide bond between ECL2 and TM3 is conserved in 92% of human GPCRs (Karnik et al., 2003), and is considered important for ligand binding and receptor activation (Woolley and Conner, 2017). In many cases, the ECL2 of GPCRs is non-structured and positioned to cover part or all of the entrance to the ligand-binding pocket (Woolley and Conner, 2017). For example, the representative protein of class A GPCRs, rhodopsin, has an ECL2 that forms a *β*-hairpin structure and is positioned deep inside the orthosteric pocket (Palczewski et al., 2000). On the other hand, the ECL2 of the *β*2-adrenergic receptor forms an *α* helix, and diversity in its structure is also observed. ECL2 is said to play an important role in ligand binding of class A GPCRs depending on its length, position, and structure (Wheatley et al., 2012). For example, when rhodopsin binds to its ligand, 11-cis-retinal, it has been reported that Ser186, Gly188, Ile189, and Tyr191 of ECL2 directly interact with the ligand (Palczewski et al., 2000). In addition, it has been reported that Arg183, Ile185, Cys186, and Asp187 of CXCR4 (C-X-C chemokine receptor type 4) are important for binding with IT1t in ECL2 (Wu et al., 2010).

The Helix encoder restricts input protein sequences to 7TM, but it is believed that performance in predicting activity can be improved by including regions outside the membrane-spanning domain, such as ECL2, in the input. Therefore, a Helix encoder model with input of both 7TM and ECL2 (TM + ECL2) was constructed, and its performance was compared to that of the conventional Helix encoder and TransformerCPI encoder. The position of ECL2 was obtained from UniProt in the same way as when it was located outside the membrane-spanning region. The architecture of the Helix encoder (TM + ECL2) was constructed by adding a block of 1D convolutional layers and GLU layers to the Helix encoder, with the new block inserted between TM4 and TM5 and adjusted to ensure that the order of all residues is maintained during position encoding. The hyperparameters and optimization functions were unchanged from those used in the Helix encoder.

Table 4 shows the AUCs for five test cases and their average AUCs. The average AUC in the test set showed that the Helix encoder (TM + ECL2) had the highest performance (Table 2, 4). Furthermore, a one-sided *t*-test with a significance level of 5% was performed for each test case of the Helix encoder and the Helix encoder (TM + ECL2), and the improvement in performance was found to be significant (*p* = 5.2*e* − 05 < 0.05). This confirms that the information from ECL2 is important for GPCR activity prediction in Class A GPCRs.

**TABLE 4:** Prediction accuracy (AUC) of Helix encoder (TM + ECL2).

Model	test_0_	test_1_	test_2_	test_3_	test_4_	Average
Helix encoder (TM + ECL2)	0.922	0.923	0.928	0.920	0.924	0.924

#### 4.1.2 Other extracellular loops

In addition to ECL2, GPCRs have other extracellular loops, namely, ECL1 and ECL3, which are located between TM2 and TM3 and TM6 and TM7, respectively. Other extracellular loops may also affect the selectivity of compounds that enter the ligand-binding pocket from the extracellular space and may also influence ligand binding. Therefore, in this section, we constructed Helix encoder (TM + ECL1) and Helix encoder (TM + ECL3) to confirm the performance of the prediction model.

The test AUCs for Helix encoder (TM + ECL1) and Helix encoder (TM + ECL3) are shown in Table 5. The average test AUCs were 0.923 and 0.921 for Helix encoder (TM + ECL1) and Helix encoder (TM + ECL3), respectively. Both models showed higher AUCs than the Helix encoder, but lower AUCs than the Helix encoder (TM + ECL2). Therefore, it was confirmed that the information from extracellular loops is important for the prediction of class A GPCRs, and the information from ECL2 contributes more to the prediction.

**TABLE 5:** Prediction accuracy (AUC) of Helix encoder (TM + ECL1) and Helix encoder (TM + ECL3).

Model	test_0_	test_1_	test_2_	test_3_	test_4_	Average
Helix encoder (TM + ECL1)	0.918	0.927	0.925	0.921	0.924	0.923
Helix encoder (TM + ECL3)	0.917	0.921	0.919	0.924	0.925	0.921

### 4.2 Attention weight analysis

**FIGURE 3:**
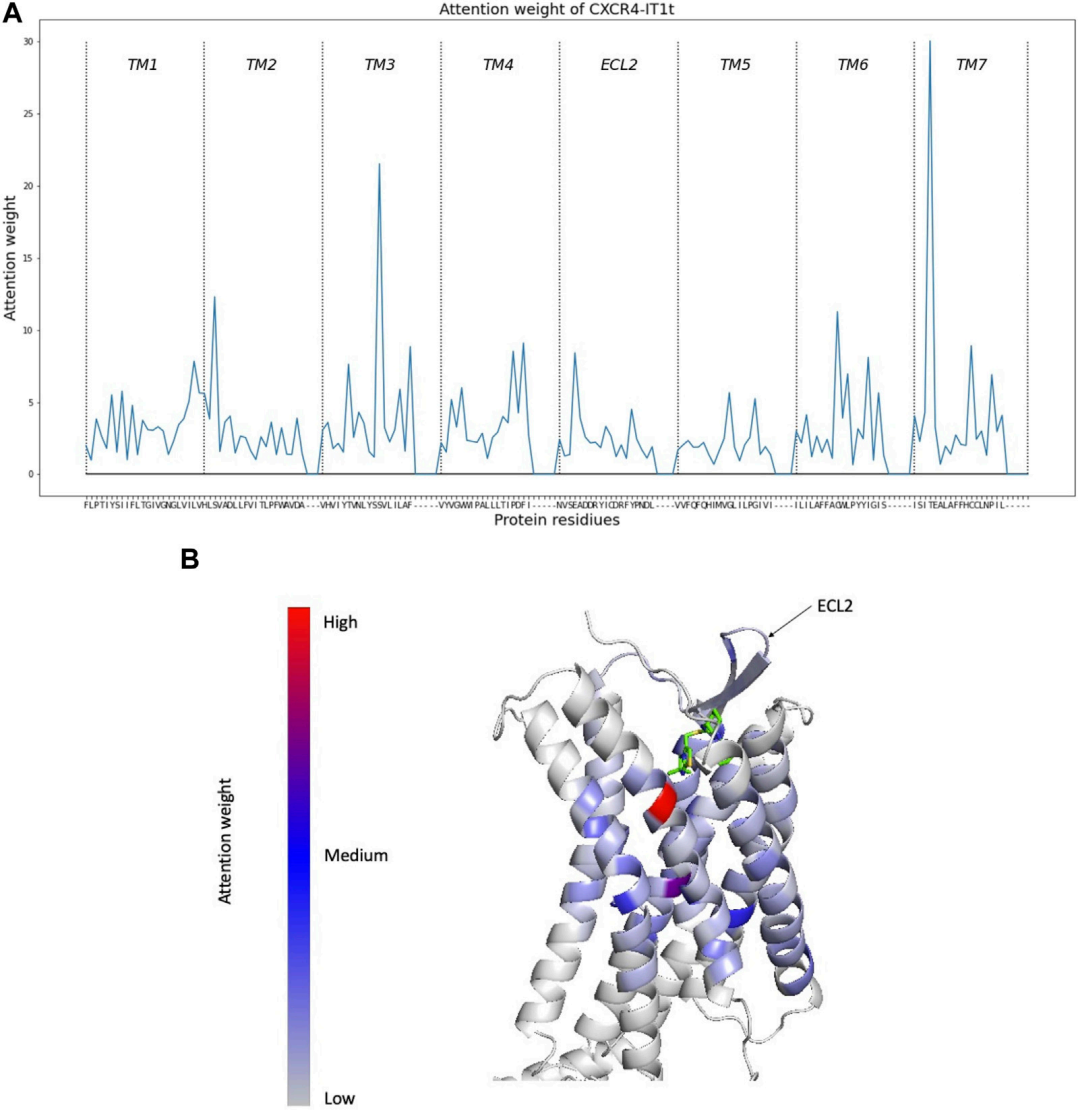
Attention weights per residue calculated by Helix encoder (TM + ECL2). **(A)** Attention weight of each region for CXCR4 and IT1t. Hyphens are padding parts. **(B)** Attention weight plotted against CXCR4 (PDB id: 3DOU, chain A).

Helix encoder (TM + ECL2) showed a higher test AUC of 0.928 in specific test split (test_2_). Thus, there may be many protein-compound pairs in test_2_ where ECL2 is considered important. To verify this hypothesis, the importance of each region (TM1, TM2, TM3, TM4, ECL2, TM5, TM6, TM7) was examined by checking how much attention weight is assigned to each region during prediction. The importance of each region was calculated based on the attention weight assigned to each residue in that region. The attention weight for each residue was calculated using the multi-headed attention of the decoder. An example of the residue-level attention weight in CXCR4 and IT1t is shown in Figure 3.

**FIGURE 4:**
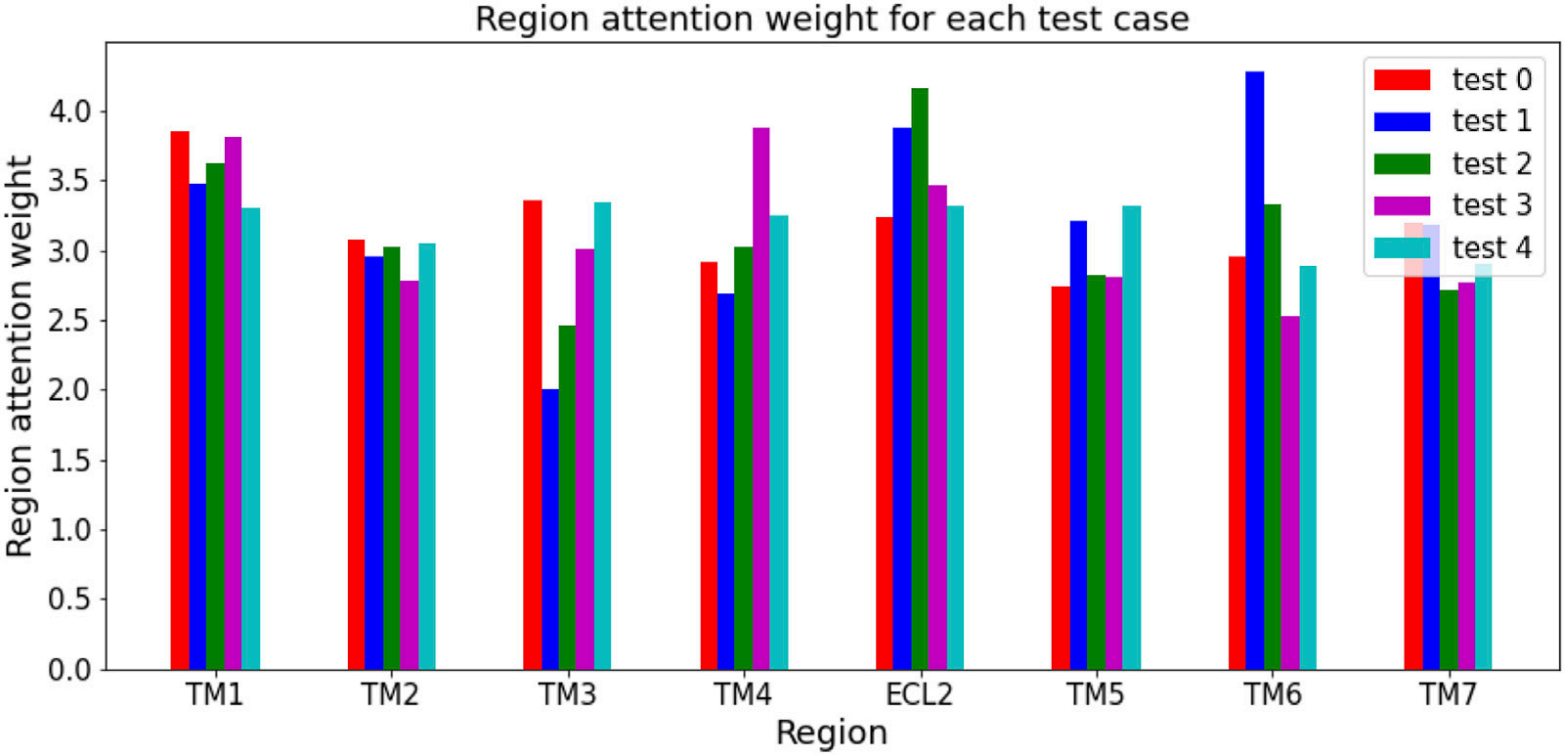
Region attention weight for each test case. The region attention weight of the ECL2 in test_2_ is higher than in the other test cases.

The region attention weight for each test case was calculated as the average of the region attention weights of protein-compound pairs in that test case. Figure 4 shows the region attention weights for the five test cases. The calculation of region attention weights for each test case revealed that test_2_ had the highest ECL2 region attention weight among the five test cases, suggesting that there may be many protein-compound pairs in test_2_ where ECL2 is considered important. Therefore, the reason why the Helix encoder (TM + ECL2) had a higher test AUC in test_2_ than other models is likely because the addition of ECL2 contributed to the prediction performance of class A GPCRs.

Whether ECL2 directly interacts with ligands or not varies not only depending on the protein, but also on the protein-compound pair. Therefore, it is a reasonable result that the use of ECL2 in the input sequence can greatly improve the performance of class A GPCR activity prediction in some cases, but only slightly in others. Since there are still many protein-compound pairs that have not been analyzed for the involvement of ECL2 in ligand binding, the introduction of ECL2 into the input sequence is important for predicting class A GPCR activity for specific protein-compound pairs. Additional experiments have shown that using not only TM but also extracellular loops leads to higher validation and test AUCs in class A GPCR activity prediction. This suggests that residues other than TM may also be important for interactions depending on the protein-compound pair, but it can be said that the information of ECL2 in particular has an impact on predictive performance based on the results of the average test AUC.

## 5 Conclusion

In this study, we developed a Helix encoder that can effectively encode class A GPCR protein sequences. The results of the performance evaluation showed that the proposed method achieved higher AUC compared to a prediction model using all protein sequences.

In this research, we used estimated transmembrane regions of a class A GPCR to improve the prediction. This process only implicitly used the structural information of a protein. However, several existing studies have investigated the ligand-GPCR interaction based on the predicted tertiary structures (Di Rienzo et al., 2022). Especially for GPCRs, specific tertiary structure prediction methods such as GPCR-I-TASSER(Zhang et al., 2015) can be used for accurate prediction, and recently AlphaFold2 has improved the availability of using modeled structures. Thus, the direct introduction of such tertiary structure information of a protein can contribute to the improvement of CPI prediction.

Furthermore, using not only transmembrane helix regions but also extracellular loops as the input, the prediction model showed better performance, especially with the addition of ECL2. Thus, it indicates that transmembrane regions and ECL2 are effective subsets of protein sequences for class A GPCR activity prediction.

After the development of TransformerCPI, several deep learning-based CPI prediction models have been proposed (Bui-Thi et al., 2022; Kurata and Tsukiyama, 2022; Qian et al., 2022). Some of them have shown better performance than TransformerCPI. In class A GPCR-compound activity prediction, the Helix encoder approach used can be substituted for the protein sequence encoders used in these studies, thereby enabling further improvement in performance.

## Data Availability

Publicly available datasets were analyzed in this study. This data can be found here: https://github.com/Haru38/HelixEncoder.
