# Supplementary material for: Helix encoder: a compound-protein interaction prediction model specifically designed for class A GPCRs
Source: Front Bioinform. 2023 May 26;3:1193025. doi: 10.3389/fbinf.2023.1193025 (PMC10250622; doi:10.3389/fbinf.2023.1193025)
Supplement: Supplementary file 1 [file DataSheet1.PDF]

Supplementary Material

1 HYPERPARAMETERS OF THE HELIX ENCODER

The details of the hyperparameters for the Helix encoder are listed in Table S1. The hyperparameters for the decoder and fully connected layers were the same as those used in TransformerCPI. In addition, RAdam was used as the optimization function and binary cross-entropy was used as the loss function.

Table S1. Hyperparameters of the Helix encoder

Name	value
Number of CNN-GLU block	3
Number of self attention block	1
Number of attention head	8
Feed foward layer inner hidden size	256
Patch size	3
Dropout	0.1
Batch size	64
Learning rate	1e–4
Weight decay	1e–4

2 TRAINING CURVE OF HELIX ENCODER AND TRANSFORMERCPI

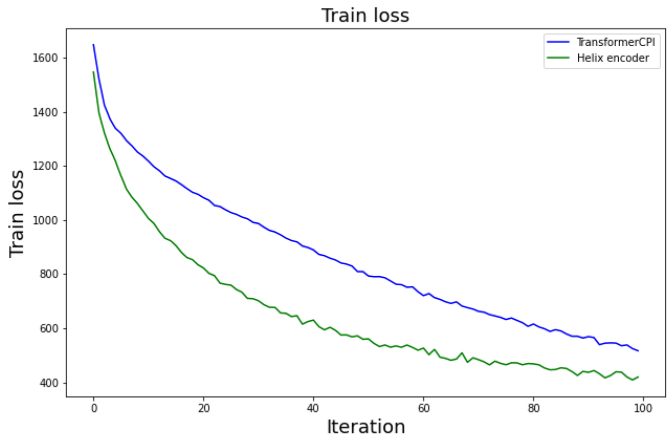

Figure S1. Training loss of Helix encoder and TransformerCPI.

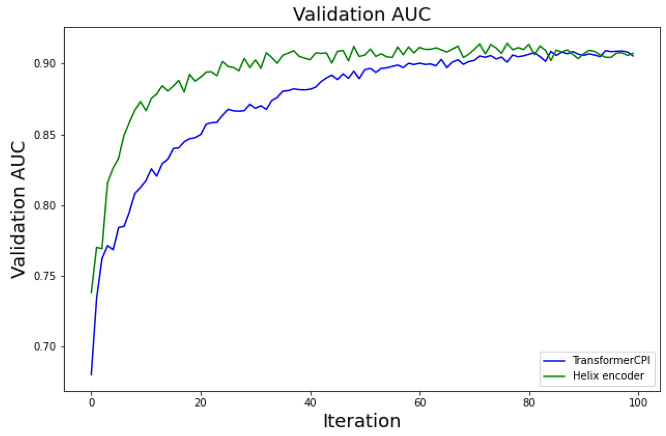

Figure S2. Validation AUC of Helix encoder and TransformerCPI.

3 EACH EXTRACELLULAR LOOP POSITION

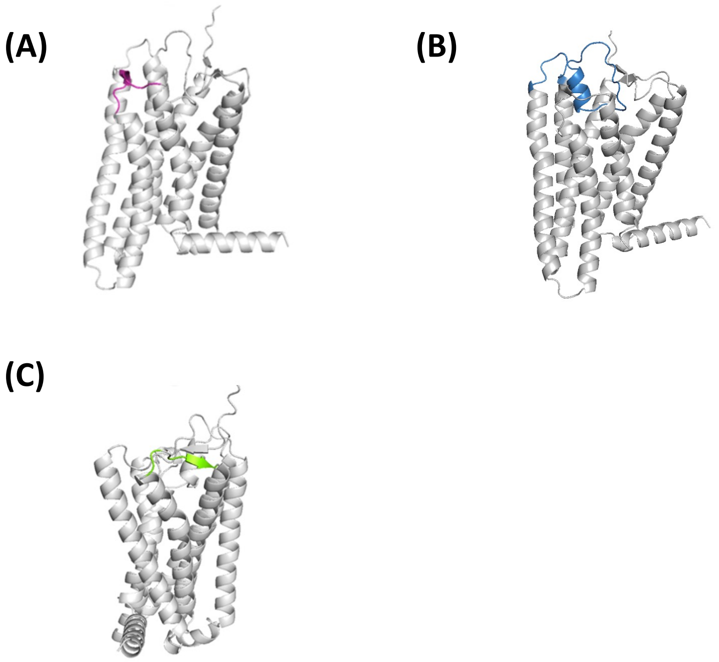

Figure S3. Each extracellular loops (UniProt ID: Q8NGI8). **(A)** Extracellular loop 1 region (yellow). **(B)** Extracellular loop 2 region (blue). **(C)** Extracellular loop 3 region (red).

4 CALCULATION METHOD FOR REGION ATTENTION WEIGHT

The importance of each region was calculated based on its attention weight. The region attention weight for each region was obtained as the average of attention weights assigned to each residue in that region. The attention weight for each residue was calculated using the multi-headed attention layer of the decoder. In this layer, the attention weight for a single head can be computed using Equation S1:

$$\mathrm{AttentionWeight} = \mathrm{softmax}\left(\frac{\mathbf{QK}^{\mathrm{T}}}{\sqrt{d_k}}\right), \tag{S1}$$

where, $\mathbf{Q}$ is the feature vector of the compound and $\mathbf{K}$ is the protein sequence vector. The region attention weight for each test case was calculated as the average of region attention weights for protein-compound pairs in that test case. For example, the region attention weight for TM1 in test_0 was calculated using Equation S2:

$$\mathrm{RegionAttentionWeight}_{\mathrm{test_0}} = \frac{1}{N_{test_0}} \sum_{i=1}^{N_{test_0}} \left(\frac{1}{L_i^{TM1}} \sum_{j=1}^{L_I^{TM1}} AW_{i,j} \right), \tag{S2}$$

where, N_{test_0} is the data size of $\mathrm{test0}$, Li^{TM1} is the length of TM1 in a certain protein contained in $\mathrm{test0}$, and AWi, j is the attention weight for a certain residue.
